# Trajectories of seasonal influenza vaccine uptake among French people with diabetes: a nationwide retrospective cohort study, 2006–2015

**DOI:** 10.1186/s12889-019-7209-z

**Published:** 2019-07-09

**Authors:** Aurélie Bocquier, Sébastien Cortaredona, Lisa Fressard, Pierre Loulergue, Jocelyn Raude, Ariane Sultan, Florence Galtier, Pierre Verger

**Affiliations:** 1 Aix Marseille Univ, IRD, AP-HM, SSA, VITROME, 19-21 Boulevard Jean Moulin, 13385 Marseille Cedex 05, France; 2 IHU-Méditerranée Infection, Marseille, France; 3 ORS PACA Observatoire Régional de la Santé Provence-Alpes-Côte d’Azur, Marseille, France; 4 INSERM, F-CRIN Innovative Clinical research Network in vaccinology (I-Reivac), GH Cochin Broca Hôtel Dieu, 75014 Paris, France; 5 Université Paris Descartes, Sorbonne Paris cité, Paris, France; 6 Inserm CIC 1417, Paris, France; 7 Assistance Publique Hôpitaux de Paris, CIC Cochin-Pasteur, Paris, France; 8 EHESP Rennes, Université Sorbonne Paris Cité, Paris, France; 9 Unité des Virus Emergents (UVE: Aix-Marseille Univ – IRD 190 – Inserm 1207 – IHU Méditerranée Infection), Marseille, France; 10 Endocrinology-Diabetology-Nutrition Department, University Hospital, Montpellier, France; 11 PhyMedExp, University of Montpellier CNRS INSERM, Montpellier, France; 12 CIC 1411 CHU Montpellier Hôpital Saint Eloi, Montpellier, France

**Keywords:** Diabetes mellitus, Influenza vaccines, Cohort studies, Administrative claims, Healthcare

## Abstract

**Background:**

Annual seasonal influenza vaccination (SIV) is recommended for people with diabetes, but their SIV rates remain far below public health targets. We aimed to identify temporal trajectories of SIV uptake over a 10-year period among French people with diabetes and describe their clinical characteristics.

**Methods:**

We identified patients with diabetes in 2006 among a permanent, representative sample of beneficiaries of the French National Health Insurance Fund. We followed them up over 10 seasons (2005/06–2015/16), using SIV reimbursement claims and group-based trajectory modelling to identify SIV trajectories and to study sociodemographic, clinical, and healthcare utilization characteristics associated with the trajectories.

**Results:**

We identified six trajectories. Of the 15,766 patients included in the model, 4344 (28%) belonged to the “continuously vaccinated” trajectory and 4728 (30%) to the “never vaccinated” one. Two other trajectories showed a “progressive decrease” (2832, 18%) or sharp “postpandemic decrease” (1627, 10%) in uptake. The last two trajectories (totalling 2235 patients, 14%) showed an early or delayed “increase” in uptake. Compared to “continuously vaccinated” patients, those in the “progressively decreasing” trajectory were older and those in all other trajectories were younger with fewer comorbidities at inclusion. Worsening diabetes and comorbidities during follow-up were associated with the “increasing” trajectories.

**Conclusions:**

Most patients with diabetes had been continuously vaccinated or never vaccinated and thus had stable SIV behaviours. Others adopted or abandoned SIV. These behaviour shifts might be due to increasing age, health events, or contextual factors (e.g., controversies about vaccine safety or efficacy). Healthcare professionals and stakeholders should develop tailored strategies that take each group’s specificities into account.

**Electronic supplementary material:**

The online version of this article (10.1186/s12889-019-7209-z) contains supplementary material, which is available to authorized users.

## Background

Because people with diabetes are at increased risk of severe complications linked to seasonal influenza [1], the World Health Organization (WHO) and many national guidelines [2–4] recommend they receive annual seasonal influenza vaccination (SIV). The SIV rate in this population is nonetheless below WHO’s target of 75% in most Western countries [5–7], especially in France (26% in 2015/16 among those < 65 years) [8].

Although SIV must be repeated annually, few cohort studies have explored the course of SIV behaviours for several consecutive years. They have found evidence for both stable SIV behaviours and behaviour shifts (e.g., stopping SIV) [9, 10], suggesting that distinct temporal patterns (trajectories) of SIV behaviour may exist. Trajectories have been studied for other significant aspects of diabetes management (e.g., glycemic control and adherence to oral hypoglycemic agents [11, 12]), but there is no literature about SIV trajectories. Identifying trajectories of SIV behaviour among people with diabetes may help identify patients on whom prevention efforts should concentrate, with tailored communication and behaviour-change strategies.

Based on reimbursement data, this article sought to: 1) identify temporal patterns (trajectories) of SIV uptake among French people with diabetes over 10 consecutive influenza seasons (2005/06 to 2015/16) and determine their prevalence; and 2) study the sociodemographic, clinical, and healthcare utilization characteristics associated with them.

## Methods

### Study design and data source

We conducted a retrospective cohort study in the Permanent Sample of Beneficiaries (Echantillon Généraliste des Bénéficiaires, EGB). The EGB, set up in 2005, is a permanent, representative, and open national random sample of 1/97th of persons currently affiliated with one of the three major national health insurance funds in France [13]. At the time of extraction (August 2017), it included 804,089 beneficiaries. For this study, we extracted data for salaried workers (including those who are retired) only (about 86% of the French population [13], covered by the French National Health Insurance Fund, NHIF) because people affiliated with the other insurance funds were only included in the EGB in 2011.

Data include age, gender, district of residence, reimbursement claims for consultations with private healthcare professionals, medical procedures (e.g., laboratory tests), drugs purchased in the community (classified by Anatomical Therapeutic Chemical (ATC) codes), and long-term illness (LTI) status, recorded by expert physicians according to the International Classification of Diseases (ICD-10). LTI status is granted to beneficiaries with long-term and costly diseases and exempts them, regardless of income level, from copayments for related medical care [14]. Since 2006, data regarding diagnoses associated with admissions to French public or private hospitals are also available.

The NHIF granted us authorization to access the EGB, in accordance with French law.

### Study population

Using our adaptation of an NHIF algorithm [8] based on LTI status, hospitalization diagnoses, and reimbursement claims for antidiabetic drugs or hemoglobin A1c (HbA1c) assays (Additional file 1: Table S1), we selected all individuals residing in mainland France identified as treated for diabetes in 2006. We followed them up over 10 seasonal vaccination campaigns. Those who died or withdrew from the NHIF during the follow-up period were censored at the start of the year of the event.

### Seasonal influenza vaccine uptake

For each individual and each SIV season *n/n + 1* (temporal statistical units in our analyses), we constructed a binary variable “SIV uptake” *(yes, no),* based on SIV deliveries (Additional file 2: Table S2) recorded between September 1 of year *n* and March 31 of year *n + 1*. Each SIV delivered in a community pharmacy is recorded in the NHIF database.

### Characteristics of the study population

To describe the diabetes type and treatments, we constructed for each year of follow-up a 5-category variable based on LTI status and reimbursement claims for antidiabetic drugs recorded during the 6 months before the start of season *n/n + 1*. Using these annual variables, we built a variable of “diabetes treatment intensification” (*yes/no*) during follow-up. “Intensification” was defined by at least one of the following modifications: from no antidiabetic drug to at least one antidiabetic drug; from only one to at least two noninsulin antidiabetic drugs or insulin; from at least two noninsulin antidiabetic drugs to insulin.

To measure comorbidities, we calculated for each year of follow-up an individual chronic condition score (ICC) based on drug deliveries according to a previously published methodology [14]. Then we built a 3-category variable describing the course of the ICC score from the first to the last year of follow-up (*decreasing*, *increasing,* or *stable*) and included it in our analysis, as a time-stable variable [15].

Each cohort member’s number of hospital stays for each year for diabetes, diabetes complications, influenza, and influenza complications was extracted, as were the numbers of visits – separately – with general practitioners (GPs), endocrinologists, and cardiologists. GPs are responsible for the management of most patients with type 2 diabetes [9] and for referral to specialists. We also extracted changes of GP during follow-up.

The NHIF sends free vaccination vouchers each season to individuals aged 65 years or older and to those patients with diabetes with an LTI status: we constructed a 3-category variable to describe receipt of this voucher (Additional file 3: Table S3). The voucher enables these patients to obtain the vaccine free of charge at the pharmacy, without a doctor’s prescription. They must then make an appointment with either a doctor or a nurse for its administration.

### Statistical analysis

We ran group-based trajectory (GBT) modelling to identify subgroups of individuals with similar patterns of SIV dispensing over time during the 10-year follow-up period. GBT modelling is a data-driven semiparametric method designed to analyze the evolution of an outcome over time and to identify, within a population with unobserved heterogeneity, distinct clusters of individuals following similar trajectories of behaviors related to this outcome [16, 17]. It makes it possible to select the model with an optimal number of distinct trajectories that most appropriately represent the heterogeneity in the population [15]. To compare the models’ goodness of fit, we used the Bayesian information criterion (BIC) and individual posterior class-membership probabilities (i.e.*,* the probability of belonging to a trajectory given the information collected). Starting with a one-trajectory solution, we added one trajectory at a time, testing each model fit and balancing it with our objective of identifying distinct and interpretable trajectories. The prevalence of each trajectory and the relevance of the solutions were also considered, as recommended by Nagin and Odgers [18]. To determine the order of polynomials for all trajectories, we started with third-degree polynomials and used the standard operating procedure, i.e. stepwise elimination of non-significant polynomial higher orders [19]. Early applications of GBT modelling have assumed that all attrition (including both loss to follow-up and mortality) is randomly distributed among all trajectories. A recent enhancement of the GBT approach enables the joint modelling of the outcome of interest and non-random missingness [20, 21]. Using this methodology, we were able to model attrition probabilities (mortality represented the vast majority of attrition in our study) jointly with the estimation of SIV trajectories.

The demographic, clinical, and healthcare utilization factors were added to the model as predictors of trajectory group membership. This joint estimation of trajectories and predictors of the probability of group membership allowed us to take into account the uncertainty in participants’ trajectory group membership [15]. We used Zhang’s correction to estimate adjusted risk ratios from the estimated ratios [22].

All statistical analyses were performed with SAS statistical software, version 9.4 (SAS Institute Inc., Cary, NC). GBT analyses were conducted with the TRAJ procedure [16].

## Results

### Study population characteristics (Table 1)

Of the 17,259 subjects with diabetes included in the cohort in 2006, 46% were women; mean age at inclusion was 65.0 ± 13.7 years. About 10% were identified with type 1 diabetes at inclusion; only 70% had LTI status for diabetes then. Over the 10-year follow-up, 31% of the initial cohort died (Additional file 4: Table S4), for a death rate of 36‰ person-years, and 3% were lost to follow-up.

**Table 1:** Study cohort characteristics during the first and last seasons *n/n + 1* of follow-up (EGB, France, 2006/07–2015/16)

	2006/07 (*n* = 17,259)	2015/16 (*n* = 11,440)^a^
	%^b^	%^b^
Sociodemographic characteristics		
Age (years) on 12.31.*n* – mean (SD)	65.0 (13.7)	70.5 (12.8)
Women	46.1	47.1
Clinical characteristics		
Type and treatment of diabetes		
Type 1 diabetes^c^	9.2	11.2
Other types -- no antidiabetic drug	15.8	9.2
Other types - only one noninsulin antidiabetic drug	35.3	22.0
Other types -- ≥ 2 noninsulin antidiabetic drugs	28.2	33.6
Other types -- insulin treatment ± antidiabetic drugs	11.5	24.0
Weighted individual chronic condition score^d^ – mean (SD)	0.8 (0.5)	0.9 (0.5)
Annual rate of hospitalization for diabetes or its complications^e^	6.2	4.3
Annual rate of hospitalization for influenza or its complications^e^	0.7	1.4
Healthcare utilization		
Annual number of consultations^f^ with -- mean (SD)		
General practitioner	8.6 (7.3)	7.9 (6.4)
Endocrinologist	0.3 (1.2)	0.4 (1.2)
Cardiologist	0.5 (1.5)	0.5 (1.4)
Change of general practitioner	4.0	10.3
Received free vaccination voucher for diabetes^g^	69.9	90.3

*SD* standard deviation

^a^Among all patients included in the cohort, 5266 (30.5%) died and 553 (3.2%) were lost during follow-up. Mean follow-up time: 8.24 ± 2.90 seasons

^b^Otherwise stated

^c^People with type 1 diabetes were those with long-term illness status for type 1 diabetes (E10 according to the ICD-10) and treated by insulin at inclusion

^d^The individual chronic condition score (ICC) was calculated as a weighted sum of 21 chronic conditions. Weights account for the severity of each condition in the score calculation (ICC range in study cohort: min = 0; max = 3.7)

^e^At least 1 hospitalization between 09.01.*n-1* and 08.31.*n*

^f^Number of consultations between 09.01.*n-1* and 08.31.*n*

^g^To identify people with diabetes, the National Health Insurance Fund uses only their Long-Term Illness (LTI) status on September 1 of each year. Nonetheless, not all patients with diabetes (especially those with diabetes other than type 1) receive the voucher, because some who should have LTI status do not apply for it

### SIV-uptake trajectories (Fig. 1)

Based on the BIC values, the fit of the models improved as the number of trajectories modeled increased. From a seven-trajectory solution and after, the prevalence of some trajectories was very low and results were difficult to interpret. Accordingly, the solution that offered the best compromise between parsimony, fit, and interpretability was a six-trajectory solution. Classification quality was good for all six (mean posterior class-membership probability > 0.82).

**Fig. 1:**
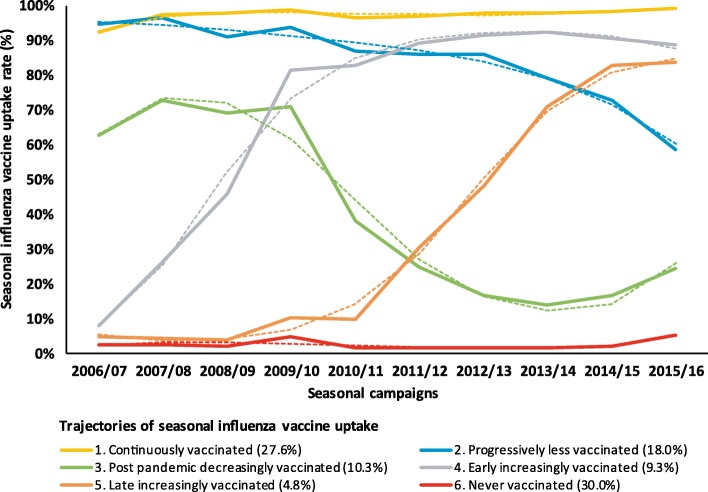
Observed (solid lines) and predicted (dashed lines) probability of seasonal influenza vaccine uptake among people with diabetes during each season of follow-up for each of six classes identified by the group-based trajectory model^a^ (EGB, France, 2006/07–2015/16, *n* = 15,766^b^). ^a^ Third-degree polynomials were used for the specifications of all trajectories, except for the “Early increasingly vaccinated” trajectory, for which a second-degree polynomial was used. ^b^ Among individuals with at least two full years of follow-up (*n* = 15,766, 90.2%), to enable calculation of two variables included in the model (i.e.*,* diabetes treatment intensification and course of weighted individual chronic condition score during follow-up)

In trajectory 1 (“continuously vaccinated”, 28% of the cohort), the SIV-uptake rate started at 92% at inclusion, then exceeded 97% throughout follow-up, with numbers of SIV injections over the 10-season follow-up ranging between 8 and 10. In trajectory 2 (“progressively less vaccinated”, 18%), SIV uptake exceeded 95% at inclusion, finally dropping to 59% in 2015/16 (range of SIV injections: [6–9]). The uptake in trajectory 3 (“postpandemic decreasingly vaccinated”, 10%) was relatively high (63–73%) and stable until the 2009/10 influenza A(H1N1) pandemic season; it dropped by 33 percentage points in 2010/11 (range of SIV injections: [2–8]). Trajectory 4 (“early increasingly vaccinated”, 9%) began with a very low SIV-uptake rate at inclusion and then immediately and rapidly increased, stabilizing around 90% in 2011/12 (range of SIV injections: [4–9]). Trajectory 5 (“late increasingly vaccinated”, 5%) looked like trajectory 4 with the increasing phase shifted forward several years (range of SIV injections: [2–6]). The individuals with trajectory 6 (“never vaccinated”, 30%) had very low SIV-uptake rates throughout follow-up (range of SIV injections: [0–2]).

### Risk factors for SIV-uptake trajectory memberships

With the “continuously vaccinated” trajectory as the reference (Table 2), the probability of belonging to the “progressively less vaccinated” trajectory was higher for individuals aged 65 years or older at inclusion, those receiving no antidiabetic drug, with high comorbidity scores at inclusion and remaining stable during follow-up, hospitalized for influenza during follow-up, and seeing GPs frequently. It was lower among women, for those with intensified diabetes treatment, seeing endocrinologists frequently, and changing GPs during follow-up.

**Table 2:** Risk factors for membership in SIV-uptake trajectories – group-based trajectory model, multinomial logistic regression^a^ (EGB, France, 2006/07–2015/16, n = 15,766^b^)

	Trajectory (ref. 1. Continuously vaccinated - *n* = 4344 (27.6%)				
	2. Progressively less vaccinated	3. Post pandemic decreasingly vaccinated	4. Early increasingly vaccinated	5. Late increasingly vaccinated	6. Never vaccinated
	*n* = 2832	*n* = 1627	*n* = 1472	*n* = 763	*n* = 4728
	18.0%	10.3%	9.3%	4.8%	30.0%
	Adjusted risk ratio [95% confidence interval]				
Sociodemographic characteristics					
Age at inclusion > 65	**2.89 [2.47;3.33]**	**0.68 [0.60;0.78]**	**0.33 [0.28;0.38]**	**0.14 [0.11;0.18]**	**0.56 [0.52;0.61]**
Women	**0.82 [0.75;0.91]**	**1.38 [1.26;1.51]**	0.99 [0.88;1.10]	0.98 [0.83;1.14]	**1.09 [1.05;1.14]**
Clinical characteristics					
Type and treatment of diabetes at inclusion					
Type 1^c^	0.99 [0.83;1.16]	1.11 [0.95;1.30]	**0.76 [0.59;0.97]**	**0.53 [0.37;0.76]**	**0.85 [0.76;0.94]**
Other types -- no antidiabetic drug	**1.18 [1.01;1.36]**	**1.61 [1.39;1.85]**	**3.04 [2.77;3.29]**	**2.10 [1.69;2.56]**	**1.62 [1.54;1.68]**
Diabetes treatment intensification^d^ during follow-up	**0.76 [0.68;0.84]**	1.04 [0.94;1.15]	**1.23 [1.09;1.39]**	**1.25 [1.06;1.47]**	0.96 [0.91;1.01]
Weighted individual chronic condition score^e^ at inclusion ≥ median	**1.16 [1.04;1.28]**	**0.87 [0.78;0.97]**	**0.77 [0.68;0.87]**	**0.81 [0.68;0.96]**	**0.75 [0.71;0.80]**
Course of weighted individual chronic condition score^e^ during follow-up					
Stable	**1.33 [1.06;1.60]**	0.81 [0.55;1.13]	0.68 [0.40;1.11]	0.83 [0.40;1.62]	**1.45 [1.33;1.57]**
Increasing	0.99 [0.90;1.08]	**0.73 [0.66;0.81]**	**1.19 [1.05;1.34]**	**1.21 [1.02;1.43]**	**0.89 [0.83;0.94]**
Hospitalized during follow-up					
For diabetes and its complications	1.05 [0.94;1.16]	**1.23 [1.11;1.37]**	1.01 [0.88;1.15]	1.02 [0.85;1.22]	1.05 [1.00;1.11]
For influenza and its complications	**1.32 [1.16;1.48]**	**1.44 [1.22;1.67]**	**1.46 [1.20;1.75]**	1.30 [0.91;1.81]	0.98 [0.87;1.09]
Healthcare utilization					
Frequent consultations during follow-up, with:					
General practitioner	**1.92 [1.76;2.07]**	1.02 [0.91;1.14]	0.95 [0.83;1.07]	**0.80 [0.66;0.96]**	**0.85 [0.80;0.90]**
Endocrinologist	**0.77 [0.65;0.91]**	**0.70 [0.59;0.83]**	**0.82 [0.69;0.97]**	**0.64 [0.49;0.83]**	**0.84 [0.78;0.91]**
Cardiologist	1.00 [0.90;1.11]	**0.79 [0.69;0.92]**	0.87 [0.74;1.01]	**0.71 [0.55;0.91]**	**0.79 [0.73;0.86]**
Change of general practitioner during follow-up	**0.73 [0.66;0.81]**	0.98 [0.89;1.08]	0.94 [0.84;1.05]	1.13 [0.97;1.31]	**0.93 [0.88;0.97]**

Reference groups. Age: “≤ 65 years”; gender: “men”; type and treatment of diabetes at inclusion: “other types -- ≥ 1 antidiabetic drug”; diabetes treatment intensification: “no”; weighted individual chronic condition score at inclusion: “< median”; course of weighted individual chronic condition score: “decreasing”; hospitalized during follow-up: “no”; consultations during follow-up: “number of consultations < median”; change of general practitioner: “no”

Boldface indicates statistical significance (*p* ≤ 0.05)

^a^Model adjusted for all variables displayed in the Table, as well as for district of residence (results not displayed): Paris region, northwest, northeast, southeast, and southwest. The variable “Received the free vaccination voucher” was not included in the model due to strong correlation with age (all people aged 65 years or older receive this voucher)

^b^Among individuals with at least two full years of follow-up (n = 15,766, 90.2%), to enable calculation of two variables included in the model (i.e.*,* diabetes treatment intensification and course of weighted individual chronic condition score during follow-up).

^c^People with type 1 diabetes were those with long-term illness status for type 1 diabetes (E10 according to the ICD-10) and treated by insulin at inclusion

^d^“Intensification” was defined by at least one of the following modifications during follow-up: from no antidiabetic drug to at least one antidiabetic drug; from only one to at least two noninsulin antidiabetic drugs or insulin; from at least two noninsulin antidiabetic drugs to insulin

^e^The individual chronic condition score (ICC) was calculated as a weighted sum of 21 chronic conditions. Weights account for the severity of each condition in the score calculation (ICC range in study cohort: min = 0; max = 3.7)

The remaining four trajectories (“postpandemic decreasingly vaccinated”, “early”/“late increasingly vaccinated”, and “never vaccinated”) shared several characteristics. The probability of belonging to these four trajectories was higher in patients receiving no antidiabetic drug at inclusion and lower in those aged 65 years or older, with more comorbidities at inclusion, and with frequent visits with specialists during follow-up. These trajectories also showed some specificities. The probability of belonging to the “postpandemic decreasingly vaccinated” trajectory was higher for women and individuals hospitalized for diabetes or influenza; it was lower for those with worsening comorbidities. The probability of belonging to the “early” or “late increasingly vaccinated” trajectories was higher for those with worsening diabetes and comorbidities during follow-up, and those hospitalized for influenza (for the “early” trajectory only); it was lower for individuals with type 1 diabetes. Finally, the probability of belonging to the “never vaccinated” trajectory was higher for women and for individuals with stable comorbidities, and lower for those with type 1 diabetes, with worsening comorbidities, frequent healthcare utilization, and changing GPs during follow-up.

## Discussion

### Key findings

Overall, this study shows remarkable inertia in behavioural patterns, with 28% of the subjects continuously vaccinated and 30% never vaccinated from 2006/07 to 2015/16. For two other trajectories, the SIV-uptake rate decreased during follow-up, either progressively (18%) or more sharply after the 2009/10 season (10%), while the SIV-uptake rate rose for the last two trajectories (accounting for only 14% of patients). Compared to “continuously vaccinated” people, those in the “progressively decreasing” trajectory were older; those in the “postpandemic decreasing”, “increasing”, and “never” vaccinated trajectories were younger than the reference category with fewer comorbidities at inclusion. The “increasing” trajectories were positively associated with the worsening of diabetes and comorbidities during follow-up.

### Strengths and limitations

The strengths of this study include its 10-year follow-up, the longest for any study examining SIV behaviours over time [6, 9, 10], and its large sample size. Moreover, our algorithm to identify patients with diabetes was more sensitive and allowed earlier identification than an algorithm based solely on LTI [8]. We used vaccine deliveries, which are more reliable than self-reported vaccination behaviour [23]. The dropout extension of the group-based trajectory modelling allowed us to control for potential selection biases due to non-random participant attrition (especially those due to mortality, Additional file 3: Table S3) [20].

We acknowledge some limitations. Vaccinations that took place during occupational medicine visits or at vaccination centres or some nursing homes that buy vaccines for their residents (fewer than 20% of all nursing homes [24]) are not recorded in the French NHIF databases. However, these limitations are unlikely to affect our results substantially as the vast majority of vaccinations in France are administered by private healthcare workers and are thus recorded in these databases [25]. As SIV behaviour varies by socioeconomic characteristics [26], our results cannot be extrapolated to population categories not covered by the NHIF (e.g., farmers, the self-employed) or the very few people without insurance; nonetheless, the NHIF covers 86% of the French population. Several socioeconomic (e.g., educational level) and clinical (e.g., diabetes complications) characteristics are not recorded in NHIF databases and therefore could not be studied. Specifically, no data about individuals’ knowledge, attitudes or perceptions towards SIV (e.g., beliefs about SIV efficacy, side effects) were available, although they are important drivers of SIV behaviours [27, 28] and thus probably differ according to trajectories.

### Interpretation of the findings

Our finding that most people with diabetes had stable SIV behaviours is consistent with results from previous qualitative [28] and quantitative studies with shorter follow-ups [9]. When health protective behaviours must be regularly repeated in stable contexts, patients’ responses to their healthcare workers’ recommendations may be performed almost automatically, without either conscious decision-making or thinking [29]. This interpretation is in line with recent advances in behavioural sciences showing that “much human behaviour is automatic, cued by environmental stimuli” [30]. We may assume that subjects continuously vaccinated were aware of their vulnerability to influenza (due to age and/or comorbidities [9]) before our follow-up began. Another hypothesis is that receiving a free voucher each year at least as early as inclusion (Additional file 3: Table S3) and regular medical consultations may foster SIV behaviours because they act as reminders [31] and the vouchers may facilitate access to the vaccine [8]. Conversely, studies show that continuously refusing SIV is often associated with attitudes of risk neutralization (e.g., comparing SI with other infectious diseases, feeling “mentally and physically” able to resist SI) [28]. Opportunities might also have been missed: we estimated that, at inclusion, 30% of patients with diabetes did not receive free vouchers because they did not benefit from LTI status. These patients can obtain a voucher from their doctor but this makes their pathway to vaccination still more complex as it requires first a doctor’s appointment to get a free vaccine voucher, then a trip to the pharmacy to pick up the vaccine, and then a second appointment for the actual injection.

Nonetheless, the shifts in SIV behaviour among distinct groups of patients suggest different underlying mechanisms of behaviour change. The characteristics of individuals in the “progressively less vaccinated” trajectory (the oldest in our cohort) may imply a progressive phasing-out of SIV among the frail elderly. This may result from doubts among patients, their relatives and/or their doctors about the benefits of SIV in the oldest populations, due to the scientific debate and its media coverage regarding SIV effectiveness and immunosenescence [32, 33]. Our results that patients in the “progressively less vaccinated” trajectory had less frequent consultations with endocrinologists and antidiabetic treatment might also suggest that diabetes itself and prevention of its complications has become a lower priority among these patients.

The “postpandemic decreasingly vaccinated” trajectory strongly echoes the fall in SIV coverage observed in most at-risk groups in France after the 2009 A(H1N1) pandemic season [8]. This drop has been linked to the controversies about the safety and effectiveness of the A(H1N1) vaccine surrounding the French mass vaccination campaign against the pandemic [8]. The overrepresentation of women in this trajectory is consistent with gender differences in vaccine hesitancy found for other vaccines [26].

Finally, our results regarding the “early/late increasingly vaccinated” trajectories suggest that adverse health events (e.g., intensification of diabetes treatment, worsening comorbidities) may foster or trigger adoption of SIV, which is in line with previous findings [9]. Finally, the percentages of subjects receiving free vouchers for the first time during follow-up rather than at baseline were highest in these trajectories (Additional file 3: Table S3). This finding suggests that offering a voucher might foster positive behaviour change [8, 31].

## Conclusions

Our results support the need for a change of the prevention paradigm from undifferentiated interventions to interventions that take the specificities of each trajectory into account. Evidence that SIV strongly decreases among frail elderly with diabetes reminds us of the importance of improving healthcare professionals’ perceptions of the benefit-risk balance of SIV. Practice guidelines could provide additional facts about SIV of the elderly, recognizing issues of immunosenescence and lower SIV efficacy at the individual level, but emphasizing its importance at the community level. Increasing the participation of patients’ relatives in patient education for chronic conditions might also be effective in enhancing the SIV uptake of both relatives and the elderly (i.e., indirect and direct protection) [34]. Other countries have chosen to vaccinate children –an important SI virus reservoir—however [3]. Our study also suggests that health events may represent critical periods during which healthcare workers might successfully address vaccine hesitancy; they should be more aware of these opportunities during patient care. Further interventional research is needed to design more effective interventions to tackle vaccine hesitancy regarding SIV. In particular, the use of tailored communication styles (e.g., presumptive or open approaches and motivational interviewing) that consider patients’ characteristics (e.g., vaccine hesitancy and educational level) deserve more research [35].

## Additional files

Additional file 1: **Table S1.** Algorithm used to identify individuals with diabetes in the study. (DOCX 46 kb)

Additional file 2: **Table S2.** List of seasonal influenza vaccines selected for the study. (DOCX 45 kb)

Additional file 3: **Table S3.** Prevalence and characteristics of trajectories identified by the six-class group-based trajectory model. (DOCX 49 kb)

Additional file 4: **Table S4.** Characteristics of cohort members who died during the follow-up period. (DOCX 48 kb)

## Data Availability

The data that support the findings of this study are available from the National Health Insurance Fund but restrictions apply to the availability of these data, which were used under license for the current study and so are not publicly available. Data are however available from the authors upon reasonable request and with permission of the National Health Insurance Fund.

## Abbreviations

BIC: Bayesian information criterion
EGB: Echantillon Généraliste des Bénéficiaires, Permanent Sample of Beneficiaries
GBT: group-based trajectory
GPs: general practitioners
ICC: individual chronic condition score
LTI: long-term illness
NHIF: National Health Insurance Fund
SIV: seasonal influenza vaccination

## References

[1] Mertz D, Kim TH, Johnstone J, Lam P-P, Science M, Kuster SP (2013). Populations at risk for severe or complicated influenza illness: systematic review and meta-analysis. BMJ.

[2] World Health Organization [Internet]. Fact sheet on influenza (seasonal) 2018 [cited 2019 may 14]. Available from: https://www.who.int/en/news-room/fact-sheets/detail/influenza-(seasonal).

[3] Rizzo C, Rezza G, Ricciardi W (2018). Strategies in recommending influenza vaccination in Europe and US. Hum Vaccines Immunother.

[4] American Diabetes Association (2018). 3. Comprehensive medical evaluation and assessment of comorbidities: standards of medical Care in Diabetes—2018. Diabetes Care.

[5] Loerbroks A, Stock C, Bosch JA, Litaker DG, Apfelbacher CJ (2012). Influenza vaccination coverage among high-risk groups in 11 European countries. Eur J Pub Health.

[6] Jiménez-Garcia R, Lopez-de-Andres A, Hernandez-Barrera V, Gómez-Campelo P, San Andrés-Rebollo FJ, de Burgos-Lunar C (2017). Influenza vaccination in people with type 2 diabetes, coverage, predictors of uptake, and perceptions. Result of the MADIABETES cohort a 7 years follow up study. Vaccine.

[7] Villarroel MA, Vahratian A (2015). Vaccination coverage among adults with diagnosed diabetes: United States. NCHS Data Brief.

[8] Verger P, Fressard L, Cortaredona S, Lévy-Bruhl D, Loulergue P, Galtier F (2018). Trends in seasonal influenza vaccine coverage of target groups in France, 2006 to 2015: impact of recommendations and 2009 influenza a(H1N1) pandemic. Euro Surveill.

[9] Verger P, Cortaredona S, Pulcini C, Casanova L, Peretti-Watel P, Launay O (2015). Characteristics of patients and physicians correlated with regular influenza vaccination in patients treated for type 2 diabetes: a follow-up study from 2008 to 2011 in southeastern France. Clin Microbiol Infect.

[10] Caille-Brillet AL, Raude J, Lapidus N, De Lamballerie X, Carrat F, Setbon M (2013). Trends in influenza vaccination behaviours--results from the CoPanFlu cohort, France, 2006 to 2011. Euro Surveill.

[11] Lo-Ciganic W-H, Donohue JM, Jones BL, Perera S, Thorpe JM, Thorpe CT (2016). Trajectories of diabetes medication adherence and hospitalization risk: a retrospective cohort study in a large state Medicaid program. J Gen Intern Med.

[12] Schwandt A, Hermann JM, Rosenbauer J, Boettcher C, Dunstheimer D, Grulich-Henn J (2017). Longitudinal trajectories of metabolic control from childhood to young adulthood in type 1 diabetes from a large German/Austrian registry: a group-based modeling approach. Diabetes Care.

[13] Tuppin P, de Roquefeuil L, Weill A, Ricordeau P, Merlière Y (2010). French national health insurance information system and the permanent beneficiaries sample. Rev Epidemiol Sante Publique.

[14] Cortaredona S, Pambrun E, Verdoux H, Verger P (2017). Comparison of pharmacy-based and diagnosis-based comorbidity measures from medical administrative data. Pharmacoepidemiol Drug Saf.

[15] Jones BL, Nagin DS, Roeder K (2001). A SAS procedure based on mixture models for estimating developmental trajectories. Sociol Methods Res.

[16] Jones BL, Nagin DS (2007). Advances in group-based trajectory modeling and an SAS procedure for estimating them. Sociol Methods Res.

[17] Verger P, Mmadi Mrenda B, Cortaredona S, Tournier M, Verdoux H (2018). Trajectory analysis of anxiolytic dispensing over 10 years among new users aged 50 and older. Acta Psychiatr Scand.

[18] Nagin DS, Odgers CL (2010). Group-based trajectory modeling in clinical research. Annu Rev Clin Psychol.

[19] Jones BL [Internet]. traj: group-based modeling of longitudinal data. 2018 [cited 2019 May 14]. Available from: https://www.andrew.cmu.edu/user/bjones/strtxmpl1.htm.

[20] Haviland AM, Jones BL, Nagin DS (2011). Group-based trajectory modeling extended to account for nonrandom participant attrition. Sociol Methods Res.

[21] Martin LG, Zimmer Z, Lee J (2017). Foundations of activity of daily living trajectories of older Americans. J Gerontol Ser B.

[22] Zhang J, Yu KF (1998). What’s the relative risk?: a method of correcting the odds ratio in cohort studies of common outcomes. JAMA.

[23] Jiménez-García R, Hernandez-Barrera V, Rodríguez-Rieiro C, Carrasco Garrido P, López de Andres A, Jimenez-Trujillo I (2014). Comparison of self-report influenza vaccination coverage with data from a population based computerized vaccination registry and factors associated with discordance. Vaccine.

[24] Agence technique de l’information sur l’hospitalisation (2013). Les coûts en établissement d’hébergement pour personnes âgées dépendantes.

[25] Institut de veille sanitaire (2001). Mesure de la couverture vaccinale en France. Bilan des outils et méthodes en l’an 2000.

[26] Rey D, Fressard L, Cortaredona S, Bocquier A, Gautier A, Peretti-Watel P, et al. Vaccine hesitancy in the French population in 2016, and its association with vaccine uptake and perceived vaccine risk–benefit balance. Euro Surveill. 2018;23. 10.2807/1560-7917.ES.2018.23.17.17-00816. 10.2807/1560-7917.ES.2018.23.17.17-00816 PMC5930729 29717693

[27] Nagata JM, Hernández-Ramos I, Kurup AS, Albrecht D, Vivas-Torrealba C, Franco-Paredes C (2013). Social determinants of health and seasonal influenza vaccination in adults ≥65 years: a systematic review of qualitative and quantitative data. BMC Public Health.

[28] Verger P, Bocquier A, Vergélys C, Ward J, Peretti-Watel P (2018). Flu vaccination among patients with diabetes: motives, perceptions, trust, and risk culture - a qualitative survey. BMC Public Health.

[29] Ouellette JA, Wood W (1998). Habit and intention in everyday life: the multiple processes by which past behavior predicts future behavior. Psychol Bull.

[30] Marteau TM, Hollands GJ, Fletcher PC (2012). Changing human behavior to prevent disease: the importance of targeting automatic processes. Science.

[31] Thomas RE, Lorenzetti DL (2018). Interventions to increase influenza vaccination rates of those 60 years and older in the community. Cochrane Database Syst Rev.

[32] Osterholm MT, Kelley NS, Sommer A, Belongia EA (2012). Efficacy and effectiveness of influenza vaccines: a systematic review and meta-analysis. Lancet Infect Dis.

[33] Beyer WEP, McElhaney J, Smith DJ, Monto AS, Nguyen-Van-Tam JS, Osterhaus ADME (2013). Cochrane re-arranged: support for policies to vaccinate elderly people against influenza. Vaccine.

[34] Yang L, Nan H, Liang J, Chan YH, Chan L, Sum RWM (2017). Influenza vaccination in older people with diabetes and their household contacts. Vaccine.

[35] Miller WR, Rollnick S (2013). Motivational interviewing: helping people change.

