# Supplementary material for: Trajectories of seasonal influenza vaccine uptake among French people with diabetes: a nationwide retrospective cohort study, 2006–2015
Source: BMC Public Health. 2019 Jul 9;19:918. doi: 10.1186/s12889-019-7209-z (PMC6617633; doi:10.1186/s12889-019-7209-z)
Supplement: Supplementary file 1 — Table S1. Algorithm used to identify individuals with diabetes in the study. (DOCX 46 kb) [file 12889_2019_7209_MOESM1_ESM.docx]

**Table S1** Algorithm used to identify individuals with diabetes in the study

| **Variable** | **Detailed criteria** |
| --- | --- |
| LTI^a^ status in 2006 | ICD-10 codes relative to diabetes mellitus (E10-E14, except E12 “Malnutrition-related diabetes mellitus”) |
| **AND/OR** |  |
| Hospitalization diagnoses in 2005 and/or 2006 | ICD-10 codes relative to diabetes mellitus (E10-E14, except E12 “Malnutrition-related diabetes mellitus”) |
| **AND/OR** |  |
| Drug reimbursement claims | ATC classes A10A (insulins and analogues) and A10B (blood glucose-lowering drugs, excluding insulins); ≥ 3 reimbursements in 2005 and/or 2006 |
| **AND/OR** |  |
| Laboratory test reimbursement claims | Hemoglobin HbA1C screening; ≥ 2 reimbursements in 2006 |

ATC: Anatomical Therapeutic Chemical; ICD: International Classification of Diseases; LTI: Long-Term-Illness.

^a^ LTI status is granted to insured persons with long-term and costly diseases and exempts them from copayments for any medical care associated with that disease, regardless of their income level.
