# Supplementary material for: Trajectories of seasonal influenza vaccine uptake among French people with diabetes: a nationwide retrospective cohort study, 2006–2015
Source: BMC Public Health. 2019 Jul 9;19:918. doi: 10.1186/s12889-019-7209-z (PMC6617633; doi:10.1186/s12889-019-7209-z)
Supplement: Supplementary file 2 — Table S2. List of seasonal influenza vaccines selected for the study. (DOCX 45 kb) [file 12889_2019_7209_MOESM2_ESM.docx]

**Table S2** List of seasonal influenza vaccines selected for the study

| **ATC code** | **Product name** |
| --- | --- |
| J07BB02 | FLUARIXTETRA SUSP INJ SER VACCIN SA 1 |
| J07BB02 | GRIPGUARD SUSP INJ 1 |
| J07BB02 | FLUARIX SUSP INJ 1 |
| J07BB02 | AGRIPPAL SUSP INJ 1 |
| J07BB02 | PREVIGRIP 1 BOITE DE 1, SUSPENSION INJECTABLE, VAC |
| J07BB02 | INFLUVAC ENFANT, 1 BOITE DE SUSPENSION INJECTABLE |
| J07BB02 | MUTAGRIP 1 BOITE DE 1, VACCIN GRIPPAL INACTIVE A V |
| J07BB02 | FLUVIRINE 1 BOITE DE 1, SUSPENSION INJECTABLE, VAC |
| J07BB02 | INFLUVAC 1 BOITE DE 1, 0,5 ML EN SERINGUE PREREMPL |
| J07BB02 | FLUARIX, 1 BOITE DE 1, SUSPENSION INJECTABLE EN SE |
| J07BB02 | VAXIGRIP SUSP INJ 1 |
| J07BB02 | INFLUVAC SUSP INJ 1 |
| J07BB02 | IMMUGRIP SUSP INJ 1 |
| J07BB02 | FLUARIXTETRA SUSP INJ SER 1 |
| J07BB02 | FLUARIX SUSP INJ SER 1 |
| J07X | TETAGRIP 1 BOITE DE 1, VACCIN TETANIQUE ET GRIPPAL |

ATC: Anatomical Therapeutic Chemical
