# Supplementary material for: Trajectories of seasonal influenza vaccine uptake among French people with diabetes: a nationwide retrospective cohort study, 2006–2015
Source: BMC Public Health. 2019 Jul 9;19:918. doi: 10.1186/s12889-019-7209-z (PMC6617633; doi:10.1186/s12889-019-7209-z)
Supplement: Supplementary file 3 — Table S3. Prevalence and characteristics of trajectories identified by the six-class group-based trajectory model. (DOCX 49 kb) [file 12889_2019_7209_MOESM3_ESM.docx]

**Table S3** Prevalence and characteristics of trajectories identified by the six-class group-based trajectory model^a^ (EGB, France, 2006/07–2015/16, n =15,766^b^)

|  | **Trajectory** | | | | | |  |  |
| --- | --- | --- | --- | --- | --- | --- | --- | --- |
|  | **1. Continuously**  **vaccinated**  **n = 4344**  **27.6%** | **2. Progressively**  **less vaccinated**  **n = 2832**  **18.0%** | **3. Post-pandemic**  **decreasingly**  **vaccinated**  **n = 1627**  **10.3%** | **4. Early**  **increasingly**  **vaccinated**  **n = 1472**  **9.3%** | **5. Late**  **increasingly**  **vaccinated**  **n = 763**  **4.8%** | **6. Never**  **vaccinated**  **n = 4728**  **30.0%** | **All**  **n=15,766** | ***p^c^*** |
| Death rate (deaths/1000 person-years) | 4.5 | 115.3 | 21.6 | 20.0 | 6.9 | 22.2 | 36.3 | <.0001 |
| **Received the free vaccination voucher**^d^ (%) | | |  |  |  |  |  | <.0001 |
| Yes, since inclusion | 97.4 | 99.3 | 92.5 | 74.1 | 63.6 | 79.6 | 88.1 | <.0001 |
| Yes, for the first time during the follow-up | 2.5 | 0.6 | 6.6 | 25.5 | 36.0 | 16.7 | 10.6 |  |
| Never during the follow-up | 0.1 | 0.1 | 0.9 | 0.3 | 0.4 | 3.7 | 1.3 |  |

^a^ Each individual has been assigned to the trajectory for which his/her class-membership probability (posterior probabilities) was the highest.

^b^ Among individuals with at least two full years of follow-up (n = 15 766, 90.2%) to enable calculation of two variables included in the model (*i.e.,* diabetes treatment intensification and course of weighted individual chronic condition score during follow-up).

^c^ Chi-squared test (ANOVA for quantitative variables).

^d^ To identify people with diabetes, the National Health Insurance Fund uses only their Long-Term Illness (LTI) status on September 1 of each year. Nonetheless, not all patients with diabetes (especially those with diabetes other than type 1) receive the voucher, because some who should have LTI status do not apply for it.
