# Supplementary material for: Trajectories of seasonal influenza vaccine uptake among French people with diabetes: a nationwide retrospective cohort study, 2006–2015
Source: BMC Public Health. 2019 Jul 9;19:918. doi: 10.1186/s12889-019-7209-z (PMC6617633; doi:10.1186/s12889-019-7209-z)
Supplement: Supplementary file 4 — Table S4. Characteristics of cohort members who died during the follow-up period. (DOCX 48 kb) [file 12889_2019_7209_MOESM4_ESM.docx]

**Table S4** Characteristics of cohort members who died during the follow-up period (EGB, France, 2006/07–2015/16)

|  | **Deceased people (n=5266)** |
| --- | --- |
|  | **%^a^** |
| **Sociodemographic characteristics** |  |
| Age (years) at death – mean (SD) | 78.6 (10.8) |
| Women | 44.6 |
| **Clinical characteristics** |  |
| Patients with newly identified diabetes (*i.e.*, no diabetes detected the year before inclusion) | 7.4 |
| Type and treatment of diabetes the year before death |  |
| Type 1 diabetes | 8.6 |
| Other types -- no antidiabetic drug | 19.2 |
| Other types - only one noninsulin antidiabetic drug | 25.3 |
| Other types -- ≥ 2 noninsulin antidiabetic drugs | 18.7 |
| Other types -- insulin treatment ± antidiabetic drugs | 28.2 |
| Weighted individual chronic condition score the year before death^b^ – mean (SD) | 1.08 (0.5) |
| Hospitalized for diabetes or its complications the year before death | 5.3 |
| Hospitalized for influenza or its complications the year before death | 3.7 |

SD: standard deviation.

^a^ Otherwise stated.

^b^ The individual chronic condition score (ICC) was calculated as a weighted sum of 21 chronic conditions. Weights account for the severity of each condition in the score calculation (ICC range in study cohort: min = 0; max = 3.7).
